# Supplementary material for: Chitosan-Hydrogel Polymeric Scaffold Acts as an Independent Primary Inducer of Osteogenic Differentiation in Human Mesenchymal Stromal Cells
Source: Materials (Basel). 2020 Aug 11;13(16):3546. doi: 10.3390/ma13163546 (PMC7475832; doi:10.3390/ma13163546)
Supplement: Supplementary file 1 [file materials-13-03546-s001.pdf]

materials

Article

Chitosan-Hydrogel Polymeric Scaffold Acts as an Independent Primary Inducer of Osteogenic Differentiation in Human Mesenchymal Stromal Cells

Simona Bernardi [1,2,*], Federica Re [1,2], Katia Bosio [1,2], Kamol Dey [3], Camillo Almici [4], Michele Malagola [1], Pierangelo Guizzi [5], Luciana Sartore [3] and Domenico Russo [1]

1 Department of Clinical and Experimental Sciences, University of Brescia, Bone Marrow Transplant Unit, ASST Spedali Civili, Brescia 25123, Italy; federicare91@gmail.com (F.R.); katia.bosio49@gmail.com (K.B.) michele.malagola@unibs.it (M.M.); domenico.russo@unibs.it (D.R.)
2 Centro di Ricerca Emato-Oncologica AIL (CREA), ASST Spedali Civili, Brescia 25123, Italy;
3 Department of Mechanical and Industrial Engineering, University of Brescia, Brescia 25123, Italy; k.dey@unibs.it (K.D.); luciana.sartore@unibs.it (L.S.)
4 Laboratory for Stem Cells Manipulation and Cryopreservation, Department of Transfusion Medicine, ASST Spedali Civili, Brescia 25123, Italy; camillo.almici@asst-spedalicivili.it
5 Orthopedics and Traumatology Unit, ASST-Spedali Civili, Brescia25123, Italy; pierangelo.guizzi@asst-spedalicivili.it
* Correspondence: simona.bernardi@unibs.it

Received: 6 July 2020; Accepted: 7 August 2020; Published:

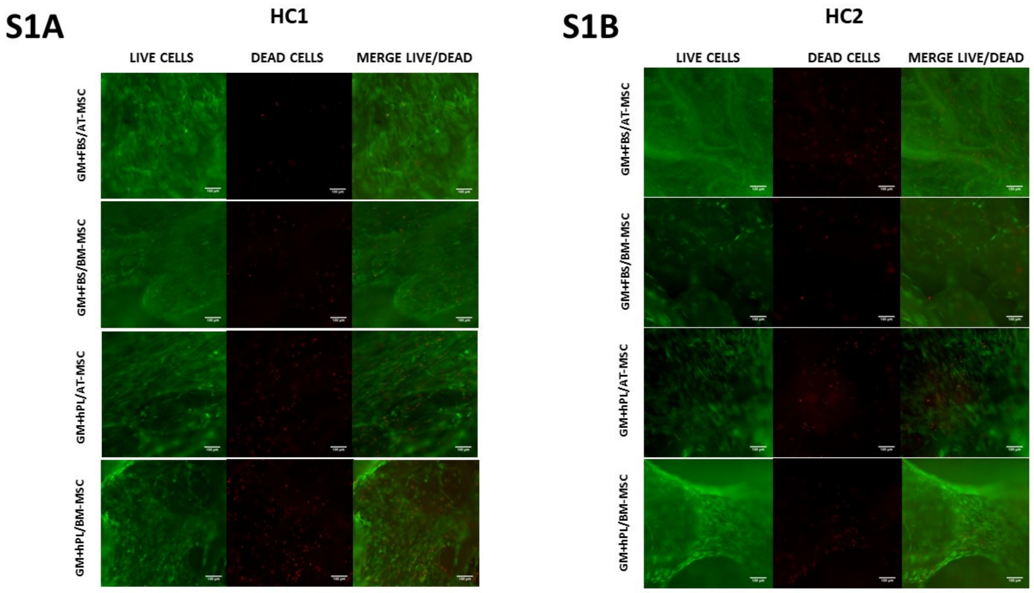

Figure 1. (A) Live/dead assay conducted after 21 days of cell culture within HC1. The Live/dead assay was performed using calcein, which converted the intracellular esterase activity of live cells, and ethidium, which enters damaged cells. The green colour shows the live cells, calcein stained, while the red cells, ethidium stained, represent the dead cells. Images were taken at 5x, with a scale-bar of 100 μm. Fluorescence analysis of whole scaffolds were made both for BM-hMSCs and for AT-hMSCs in all conditions. **(B)** Live/dead assay conducted after 21 days of cell culture within HC2. The Live/dead assay was performed using calcein, which converted the intracellular esterase activity of live cells, and ethidium, which enters damaged cells. The green colour shows the live cells, calcein stained, while the red cells, ethidium stained, represent the dead cells. Images were taken at 5x, with

a scale-bar of 100 μm. Fluorescence analysis of whole scaffolds were made both for BM-hMSCs and for AT-hMSCs in all conditions.

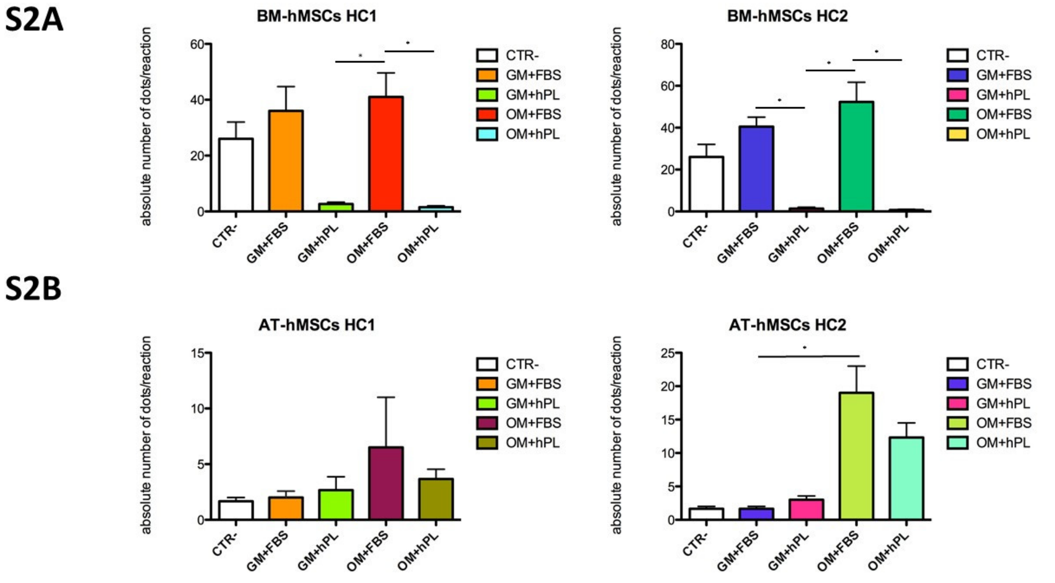

Figure 2. (**A**) The Figure reports the absolute quantification of aggrecan transcript level obtained by dPCR analysis on BM-hMSCs at 21 days. All conditions are reported: GM+FBS, GM+hPL, OM+FBS, OM+hPL, for HC1. GM+FBS, GM+hPL, OM+FBS, OM+hPL, for HC2. CTR- obtained by 2D culture of BM-hMSCs cultured with GM served as negative control. Data are expressed as absolute number of positive dots/reaction. Statistical significances calculated by t-test with Welch's correction (*p≤ 0,05). (**B**) The Figure reports the absolute quantification of aggrecan transcript level obtained by dPCR analysis on AT-hMSCs at 21 days. All conditions are reported: GM+FBS, GM+hPL, OM+FBS, OM+hPL, for HC1. GM+FBS, GM+hPL, OM+FBS, OM+hPL, for HC2. CTR- obtained by 2D culture of BM-hMSCs cultured with GM served as negative control. Data are expressed as absolute number of positive dots/reaction. Statistical significances calculated by t-test with Welch's correction (*p≤ 0,05).

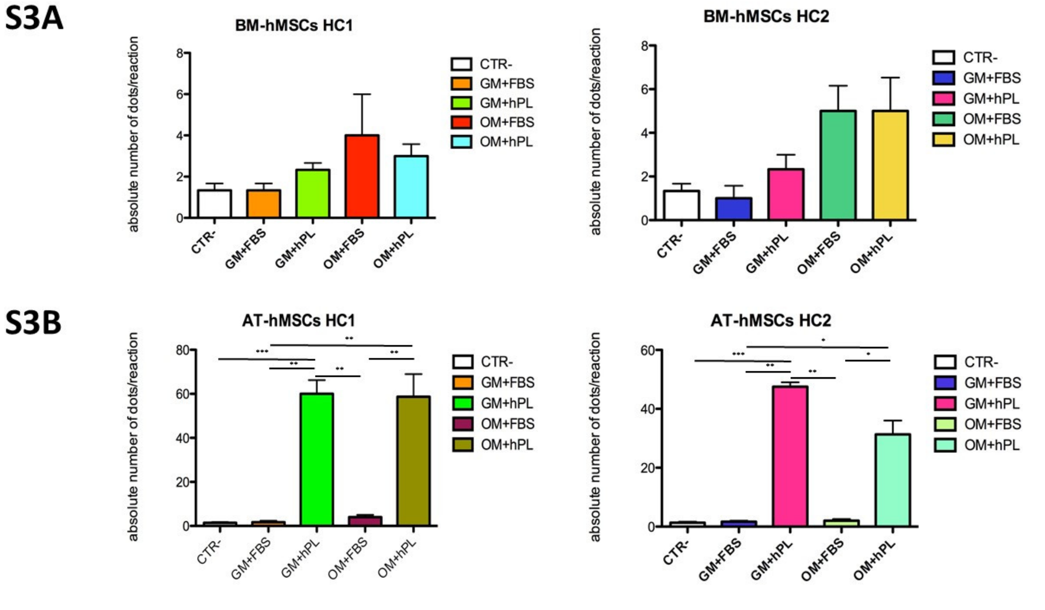

Figure 3. (**A**) The Figure reports the absolute quantification of *FABP4* transcript level obtained by dPCR analysis on BM-hMSCs at 21 days. All conditions are reported: GM+FBS, GM+hPL, OM+FBS,

OM+hPL, for HC1. GM+FBS, GM+hPL, OM+FBS, OM+hPL, for HC2. CTR- obtained by 2D culture of BM-hMSCs cultured with GM served as negative control. Data are expressed as absolute number of positive dots/reaction. Statistical significances calculated by t-test with Welch's correction. No statistical difference was observed (**B)** The Figure reports the absolute quantification of FABP4 transcript level obtained by dPCR analysis on AT-hMSCs at 21 days. All conditions are reported: GM+FBS, GM+hPL, OM+FBS, OM+hPL, for HC1. GM+FBS, GM+hPL, OM+FBS, OM+hPL, for HC2. CTR- obtained by 2D culture of BM-hMSCs cultured with GM served as negative control. Data are expressed as absolute number of positive dots/reaction. Statistical significances calculated by t-test with Welch's correction (*p≤ 0,05, **p≤ 0,01, ***p≤ 0,001).

© 2020 by the authors. Submitted for possible open access publication under the terms and conditions of the Creative Commons Attribution (CC BY) license (http://creativecommons.org/licenses/by/4.0/).
